# Behavioral and psychological symptoms of dementia: A study of symptomatology

**DOI:** 10.4103/0019-5545.44903

**Published:** 2009

**Authors:** S. Shaji, Srija Bose, Shan Kuriakose

**Affiliations:** Department of Psychiatry, Bethsada Hospital, Vengola. P.O, Perumbavoor, Kerala, India; 1 Dementia Care Centre, Palarivattom, Kochi, India

**Keywords:** Behavioral and psychological symptoms, dementia Dementia, Alzheimer's Disease, Behavioral symptoms in dementia

## Abstract

**Background:**

Behavioral disturbances are integral to the dementing process and contribute adversely to the quality of life of the patients and caregivers. Information regarding the nature of symptoms in Alzheimer's disease is limited in the Indian context.

**Aim:**

To study the nature of symptoms in Alzheimer's disease using the Malayalam adaptation of Behavioral Pathology in Alzheimer's disease Rating Scale (BEHAVE-AD).

**Materials and Methods:**

Forty patients diagnosed as having Alzheimer's disease as per DSM-IV criteria were studied using the Malayalam version of BEHAVE-AD.

**Results:**

Delusions and paranoid ideations were present in 53% of the sample studied and 33% had hallucinations. Activity disturbances were seen in 65%, aggressive symptoms in 75% and diurnal rhythm disturbances in 55%. Affective disturbances and anxiety symptoms were present in 37% and 43% respectively.

**Conclusion:**

The prevalence of behavioral and psychological symptoms of dementia was found to be high.

## INTRODUCTION

Dementia is emerging as a major public health problem in India along with the demographic transition towards aging. The symptoms of dementia can be categorized in to three major domains: cognition, behavior and impairment in functioning related to activities of daily living. Behavioral disturbances are integral to the description of dementing disorders.[1] Behavioral and psychological symptoms of dementia (BPSD) can be defined as a heterogeneous range of psychological reactions, psychiatric symptoms and behaviors occurring in people with dementia of any etiology.[2] The significance of BPSD is that it can result in premature institutionalization, increased cost of care, emotional suffering for the patients and caregiver.[3–5] It is associated with increased morbidity and mortality to the patients and increased burden of care.[6,7] These symptoms are amenable to various modalities of treatment.[8]

The basis of diagnosis of individual components that make up BPSD revolves around a clinical interview, direct observation of a person with dementia, or proxy report from a carer or other observer. The three approaches often need to be combined, and some assessment scales rely more on one approach than on another.[2] Behavioral Pathology in Alzheimer's Disease Rating Scale (BEHAVE-AD)[9] is a 25 item rating scale with assessments on a 4-point severity scores. It was specifically designed to assess behavioral and psychological symptoms in dementia that would be remediable to both psychological and pharmacological interventions. BEHAVE-AD was completed by interviewing an informant who was usually a primary caregiver. The scale has been validated and is widely used particularly in clinical trials.[10] Increased international collaboration in clinical trials has created a need for scale translations and cross-culturally valid instruments. The use of scales that have been poorly translated and translations that have not been validated can lead to erroneous results.[11]

Aims of the study are as follows:

1. To prepare a Malayalam version of Behavior Pathology of Alzheimer's disease Rating Scale (BEHAVE-AD).
2. To analyze the behavioral and psychological symptoms in Alzheimer's disease.

## MATERIALS AND METHODS

The sample consisted of forty consecutive cases utilized the services from a Dementia care center, and the Dementia care unit of a psychiatric hospital. All patients met DSM-IV criteria for dementia of Alzheimer's type. A score of 4 or less in the Hachinski Ischemic Scale[12] was used to exclude cases with vascular dementia. The clinical staging of dementia was done by the Global Deterioration Scale (GDS).[13] The clinical evaluation of these patients included detailed medical, psychiatric and neurological histories and examinations. The relevant laboratory investigations and neuroimaging were done to rule out other possible causes of dementia. The primary caregivers of patients were interviewed using the Malayalam adaptation of BEHAVE-AD.

## BEHAVE-AD: Malayalam adaptation

The original tool was translated in to Malayalam by two psychiatrists and a psychologist independently. These translations were pooled together, and a final version was prepared. The Malayalam version was then translated in to English. The translation-retranslation reliability was found to be satisfactory. The Malayalam version was then used for the study.

Difficulties were experienced while translating terms such as “delusion” “imposter” “infidelity” and “paranoia.” There are no commonly used equivalent terms in Malayalam for these words. For example, the term delusion was translated as false belief though it did not contain the exact conceptual meaning of the term “delusion.” But it is the most appropriate term that could be used. Though there were no equivalent terms for “imposter,” paranoia” and “infidelity” in Malayalam, the ideas were expressed in a descriptive manner. The item related to institutionalization did not seem relevant in the Indian cultural context as such practices are rare in our country. The examples given for purposeless activities such as opening and closing of pocket book were not relevant as it is not a common practice with our elderly to keep pocket book. Example for inappropriate activity “like empty plates in the oven” was not suited to our situation so they were deleted.

Of the 40 cases, 20 cases were selected for the reliability study. The reliability of the tool was assessed using inter-rater method. The inter-rater reliability was determined between ratings of three raters consisting of a psychiatrist, psychologist and a social worker. All the persons were interviewed by one of the raters and other raters were simultaneously present during the time of interview. The interviewer was rotated across the persons and all the raters made the ratings independently.

The statistical analysis was performed using the Statistical Package for Social Sciences (SPSS). The findings were described in terms of percentages. The correlations were tested using Pearson correlation analysis.

## RESULTS

The sample characteristics are given in Table 1.

**Table 1:** Socio-demographic characteristics of the sample

Sample characteristics	*N* = 40
Gender	
Male	17 (42.5%)
Female	23 (57.5%)
Age range	68-94
Mean age (s.d):	84.5 (s.d. = 6.8)
Education	
Illiterate	6 (15%)
Primary	17 ((42.5%)
Secondary	10 (25%)
College	7 (17.5%)
Religion	
Hindu	15 (37.5%)
Christian	18 (45%)
Muslim	7 (17.5%)
Marital status	
Unmarried	5 (12.5%)
Married	14 (35%)
Widow	16 (40%)
Widower	5 (12.5%)

Six patients belonged to GDS stage-4, 16 to GDS stage-5 and 20 to GDS stage-6. Eight people had previous consultations with neurologists or psychiatrists, but were on irregular treatment and their symptoms were not under control.

The inter-rater reliability for each of the seven subgroups of symptoms varied between 0.70 and 0.92.The inter-rater reliability for the total score of part-1 (symptomatology) was 0.89, and for the part-2, the global rating was 0.70

Of the forty patients with Alzheimer's disease, 21 (53%) and 13 (33%) had hallucinations. Various activity disturbances such as wandering, purposeless and inappropriate activities were seen in 26 people (65%). Aggressive symptoms were present in 30 (75%) patients. Twenty-two (55%) of the 40 patients had diurnal rhythm disturbances. Affective symptoms were present in 15 (38%) and anxiety symptoms in 17 (43%) patients. The specific symptoms in each category are given in Table 2.

**Table 2:** Behavioral and psychological symptoms of dementia

Symptomatology	*N* = 40	%
Delusions and paranoid ideations	
People are stealing things	12	30
One's house is not one's own	14	35
Spouse (caregiver) is an imposter	6	15
Delusion of abandonment	2	5
Delusion of infidelity	2	5
Suspiciousness/paranoia other than above	2	5
Delusion other than above	2	5
Hallucinations	
Visual hallucination	9	23
Auditory hallucination	6	15
Olfactory hallucination	0	0
Tactile hallucination	0	0
Other hallucinations	0	0
Activitiy distrbances	
Wandering	21	52.5
Purposeless activity	19	47.5
Inappropriate activity	18	45
Aggressiveness	
Verbal out bursts	23	57.5
Physical threats/violence	16	40
Agitation	18	45
Diurnal rhytham disturbances	
Day/night disturbances	22	55
Affective disturbances	
Tearfulness	11	27.5
Depressed mood	9	23
Anxities and phobias	
Anxiety regarding upcoming events	13	32.5
Other anxieties	8	20
Fear of being left alone	6	15
Other phobias	8	20

## DISCUSSION

Culture can affect both individual and societal attitudes towards Dementia. Language, social customs, traditions, and quality and quantity of education are cultural variables relevant to scientific research in Alzheimer's disease and its behavioral and psychological symptoms. Culture can also influence BPSD and care givers response to such symptoms.[14] Almost all the patients with Alzheimer's disease develop noncognitive neuropsychiatric symptoms at same period during their course of illness.[15] Lifetime risk of neuropsychiatric disturbances is nearly 100%.[16] Resiberg *et al.*[9] identified seven major categories of behavior domain symptoms in AD. These symptoms include paranoid and delusional symptoms, hallucinatory disturbances, activity disturbances, aggressive symptoms, affective disturbances and anxieties and phobias.

Evidence is growing that the psychosis of AD is a distinct syndrome different from other common late life psychosis.[2] Between 10% and 73% of patients with AD experience delusions, with paranoid delusions being the most common.[17] Delusions and paranoid ideations were observed in 53% of the sample studied. The delusion of “One's house is not one's own” was observed in 35% of the sample studied followed by “people are stealing things” (30%). Resiberg *et al.*[9](1987) reported “people are stealing things” as the commonest type of delusion seen in AD patients (48%) followed by “One's house is not one's own” (21%). In a study conducted in India, Khandelwal(1992)[18] reported that the delusion of “people are staling things” was the commonest (27%) followed by “One's house is not one's own” (10%).

Hallucinations occur in 12% to 53% of patients with AD. Visual hallucinations are most common followed by auditory hallucination.[19] Thirteen out of forty patients (33%) had hallucinations. Twenty-three percent of the sample studied had visual hallucination and 15% had Auditory hallucinations. In a study on 178 Alzheimer's patients, 17% had hallucinations at some time, visual being slightly more common than auditory.[19] Khandelwal[18] reported Visual hallucinations in 20% of the sample studies, and Resiberg *et al.*[9] observed visual hallucinations in 12% of the sample.

Various activity disturbances were seen in 65% of the sample studied. Wandering was seen in 68% of the people and was reported to be one of the most distressing symptoms Contributing to the care burden. Purposeless activity was seen in 50% and various inappropriate activities could be seen in 45% of the patients. Khandelwal *et al.*[18] reported wandering in 23%, purposeless activity in 43% and inappropriate activity in 40%. Wandering was reported in 3 to 26% of outpatients of Alzheimer's disease.[3]

Aggressive symptoms such as verbal outbursts, physical threats or violence and agitation were observed in 75% of the patients. Fifty-eight percent showed verbal out bursts, and 40% of the sample showed physical threat or violence; agitation was seen in 45%.

Agitation is common in dementia and has a marked impact on care givers.[20] The frequency of agitation in patients with AD has been reported between 24% and 48%.[3,9] Physical violence and hitting occurring in 30% of the patients with dementia of Alzheimer's type.[9,21]

Fifty-five percent of the sample studies had diurnal rhythm disturbances. Khandelwal *et al.*[18] reported sleep disturbances in 40% of the sample studied, and Resiberg *et al.*[9] reported it in 42%.

Cross-sectional studies reported rates of depression in dementia between 1.5% and 28% and higher rates for depressive symptoms between 0% and 87%.[22–24] This variation in the prevalence of depression or depressive symptoms is mainly related to differences in diagnostic criteria, and many researchers seem to apply varying criteria in different ways.[25] Affective symptoms were present in 37% of the sample studied. Tearfulness was present in 28% and depressed mood in 23%. Khandelwal[18] reported depressive mood in 33% and tearfulness in 28% of the patients. Depression is commonly overlooked in dementia patients as it does not have a typical presentation - often there is a lack of sad or depressed affect or mood. The aphasic patient is unable to articulate the subjective experience of being depressed.[26]

Anxiety symptoms in dementia can be seen as an expression of stress in predisposed person who becomes aware of their cognitive decline. Cummings *et al.*[27] reported a 60% incidence of anxiety symptoms in AD patients. In the present study, 42.5% had anxiety symptoms, 32.5% had anxiety regarding upcoming events, 20% had other anxieties and 15% had the fear of being left alone and 20% had other phobias. Although the prevalence of anxiety disorders in community living patients with dementia is unknown, currently available study of AD patients suggests that prevalence of this symptom is quite high. As expected, this prevalence appears to vary substantially from setting to setting.[28]

The sample of the study was selected patients who utilized services from a dementia care unit a psychiatric hospital with a dementia care unit. Therefore, the generalization of the findings can be difficult. Some of the psychiatric symptoms related to sensory misinterpretations such as believing events in the television to be real and failing to recognize self in the mirror are not represented in the scale.

## CONCLUSION

Behavioral and psychological symptoms of dementia are gaining importance in clinical practice and research. BPSD is the commonest cause for medical help seeking in most of the developing countries. However, many aspects of BPSD remain unexplored. The growing interest in the BPSD is reflected in the substantial increase in the controlled clinical trials for BPSD. There exists a lot of domains of research in relation to BPSD, which include epidemiology, etiology, phenomenology and both pharmacological and nonpharmacological interventions. The research on BPSD can lead us to issues related to prevention, early intervention and overall treatment effectiveness.[29]
